# Treatment Efficacy of Semantic Feature Analysis in Logopenic and Semantic Variants of Primary Progressive Aphasia

**DOI:** 10.3390/healthcare14020272

**Published:** 2026-01-21

**Authors:** İbrahim Can Yaşa, İlknur Maviş, Tuğba Kaya

**Affiliations:** 1 Speech and Language Therapy Department, Faculty of Health Sciences, Bahcesehir University, Istanbul 34353, Turkey; 2 Division of Psychology and Language Sciences, University College London (UCL), London WC1H 0AP, UK; 3 Association of Speech and Language Therapists, Ankara 06550, Turkey; ilknur.mavis@gmail.com; 4 Speech and Language Therapy Department, Faculty of Health Sciences, University of Health Sciences, Istanbul 34668, Turkey; tugba.kaya1@sbu.edu.tr

**Keywords:** primary progressive aphasia, logopenic, semantic feature analysis treatment, language skills, quality of life

## Abstract

**Background/Objectives**: Primary Progressive Aphasia (PPA) is a neurodegenerative disorder characterized by gradual and progressive deterioration of speech and language abilities. Speech and language therapy is considered an important intervention to slow decline and support the recovery of linguistic functions in individuals with PPA. This study aims to examine the effectiveness of an elaborated Semantic Feature Analysis (SFA) approach in enhancing naming abilities and semantic networks in individuals with the logopenic and semantic variants of PPA. **Methods**: Fourteen participants were recruited, including seven individuals with logopenic PPA and seven with semantic PPA. All participants received an elaborated SFA intervention twice weekly for four weeks. The Aphasia Language Assessment Test (ADD), the Turkish Picture Naming Test (T-RAT), and the SAQOL-39 were conducted at the following three time points: prior to treatment (pre-test), immediately after treatment (post-test), and one month post-treatment (follow-up). **Results**: Significant improvements were observed in ADD, T-RAT, and SAQOL-39 scores in both logopenic and semantic PPA groups following treatment (*p* < 0.05). Although follow-up scores declined compared to posttest performance (*p* < 0.05), several follow-up scores remained higher than pretest levels. Between-group comparisons indicated no significant difference in ADD scores; however, logopenic PPA participants demonstrated higher T-RAT scores (*p* < 0.05), while semantic PPA participants showed higher SAQOL-39 scores, except at follow-up (*p* < 0.05). **Conclusions**: Preliminary results suggest that the elaborated SFA intervention is effective in improving naming skills, language functioning, and quality of life in both logopenic and semantic variants of PPA. Although treatment gains partially decreased after one month, many improvements were maintained above baseline, supporting the clinical value of SFA in managing language decline in PPA.

## 1. Introduction

Primary progressive aphasia (PPA), an insidious onset neurodegenerative disease, usually occurs between the ages of 60 and 70 [1]. It accounts for approximately 20% of all dementia cases [2]. There is usually a progressive deterioration in speech and/or language function during at least the first two years. However, behavioral function and activities of daily living, memory skills, reasoning, visuospatial processing, and social abilities are relatively preserved during this time [3,4]. Even if these cognitive and behavioral functions are impaired, language skills continue to be the most impaired area [4,5].

Three different variants of PPA have been defined, including semantic (svPPA), nonfluent/agrammatic (nfvPPA), and logopenic (lvPPA), by determining both histopathological and clinical diagnostic criteria [3]. In svPPA, anomia and comprehension deficiencies are observed, while fluent speech production and repetition abilities are preserved [6]. Histopathologically, svPPA cases are usually associated with anterior temporal lobe atrophy/hypometabolism (left greater than right) [7,8]. In nfvPPA, agrammatism and effortful speech are evident, but conceptual knowledge and single-word comprehension are relatively intact [9,10]. When patients are asked to choose between alternatives such as yes/no or he/she, they often give the wrong answer, which they then self-correct [11]. In nfvPPA, there are abnormalities in the left posterior frontal and insular cortex [12,13,14]. In lvPPA, spontaneous speech, word retrieval, and sentence repetition skills are impaired, while conceptual knowledge and comprehension skills are relatively preserved [15,16]. In addition, motor control for speech is preserved, and agrammatism is not observed [3]. Histopathologically, left posterior perisylvian temporo-parietal atrophy/hypometabolism is evident in the logopenic variant [17,18,19].

PPA will be identified with greater accuracy and frequency as the diagnostic criteria are more clearly defined. Therefore, the search for treatment for these patients has increased in recent years. Speech and language disorders due to stroke have been known for many years, and the benefits of behavioral intervention are well defined [20]. However, there are not many studies investigating the effectiveness of behavioral intervention in PPA [21,22,23,24]. Regarding behavioral interventions, the World Health Organization has grouped all non-pharmacological interventions under the following three headings: impairment, activity, and participation [25]. Impairment-based interventions are defined as interventions aimed at relieving or improving symptoms. In this intervention, the aim was to relearn words and sentences and to improve reading and writing. Activity and participation-based interventions, on the other hand, aim to resume any activity and participate in life activities (daily activities).

Word-finding difficulties and naming problems are defining features of aphasia common to all PPA subtypes [26]. Consequently, most therapeutic interventions for PPA have focused on the treatment of naming problems through specific approaches targeting semantic, phonological, or orthographic deficits. Semantic therapies, such as Semantic Feature Analysis (SFA), aim to strengthen the semantic network surrounding a target word, thereby facilitating lexical retrieval through spreading activation. Studies utilizing SFA in PPA have demonstrated improvements in naming accuracy for treated items. Conversely, phonological interventions focus on the sound structure of words, often aiding patients with logopenic variants who struggle with phonological loop deficits. Orthographic approaches, utilizing written cues, support patients by bypassing impaired phonological or semantic routes. While each approach has shown efficacy, semantic-based treatments remain particularly prominent for addressing the core anomia found in semantic and logopenic variants [21,22,27]. Although fewer studies have focused specifically on the treatment of anomia in patients with lvPPA compared to other variants, existing research has consistently demonstrated positive treatment outcomes using these strategies [17,28,29]. Results from studies suggest that behavioral therapies on their own may be moderately effective [15,24,30,31]. Studies have shown that various speech and language therapy methods, such as target picture naming, naming categories in a limited time window, and error-free learning, can preserve naming skills [17,32,33]. Indeed, it has been determined that behavioral language therapy studies performed in patients with PPA slow the progression of language deficiencies, usually limited to the field of application [12,34]. However, due to the degenerative nature of PPA, intervention gains are predicted to decline over time [35].

Semantic feature analysis (SFA) was first described by Ylvisaker and Szekeres for activating semantic networks [36,37]. This treatment is based on lexical retrieval models that envision the semantic system as a network of concepts. In lexical access models, trying to name a pictorial object also activates the properties of that object [38,39]. The network of concepts activated as a result is spread over semantically related lexical items. However, the lexical item with the highest activation is selected. The phonological representation associated with the selected word item is activated, and a motor program drives the production of the name [40]. There is more evidence that significant improvements have been found in the retrieval of treated words and that they can be sustained than that of the retrieval of untreated words [41,42,43,44].

In summary, there is no specific speech and language intervention recommended for patients with PPA. However, research examining speech and language interventions has shown that targeted therapy approaches, which aim to improve naming difficulties and also language production, particularly approaches such as SFA, can benefit both svPPA and lvPPA patients [41,42,45]. Accordingly, the study aims to examine the effectiveness of an elaborated Semantic Feature Analysis (SFA) approach in enhancing naming abilities and semantic networks in individuals with the logopenic and semantic variants of primary progressive aphasia (PPA).

## 2. Materials and Methods

### 2.1. Participants

This is a group study that included individuals (i) diagnosed with PPA as a result of language evaluation and neurological examinations (MR and PET scans) and (ii) native Turkish speakers in one calendar year. The patients included in the study were diagnosed with PPA in accordance with the consensus criteria of Gorno-Tempini et al. (2011) [3]. It was announced that free evaluation and therapy would be provided for individuals with PPA diagnosed with logopenic and semantic variant and their families (through the relevant clinics where the diagnosis was made). The details of the study were shared with the participants, who then communicated by phone or e-mail and were informed about the evaluation process and the content of the study. Data collection was conducted between June and September 2024, and participants were recruited consecutively based on their admission order. A total of 19 individuals with PPA were screened for eligibility. Of these, five were excluded as follows: three due to the intensity of the therapy processes and follow-up difficulties, one because they only sought evaluation, and one due to a stroke occurrence one week prior to the study. Consequently, 14 participants were enrolled, and all completed the intervention and follow-up assessments; no dropouts occurred. Three of them were not included in the study because of the intensity of the therapy processes and difficulties in follow-up, one because they only came for evaluation and advice, and one participant had a stroke one week before the study started. The study included 7 semantic variant and 7 logopenic variant patients. Demographic (Table S1) and clinical details of the 14 participants included in the study are given in Table 1.

The criteria for inclusion in the research were determined as being at least a high school graduate, being between the ages of 40 and 65, being able to understand the instructions given, and not being diagnosed with additional neurological disease. Exclusion criteria comprised severe sequelae secondary to previous neurological conditions; age below 40 or above 65 years; history of psychoactive substance use other than tobacco; diagnosed developmental disorders; history of head trauma associated with loss of consciousness; and any history of brain tumor, neurosurgical intervention, or intracranial implantation.

**Figure 1:**
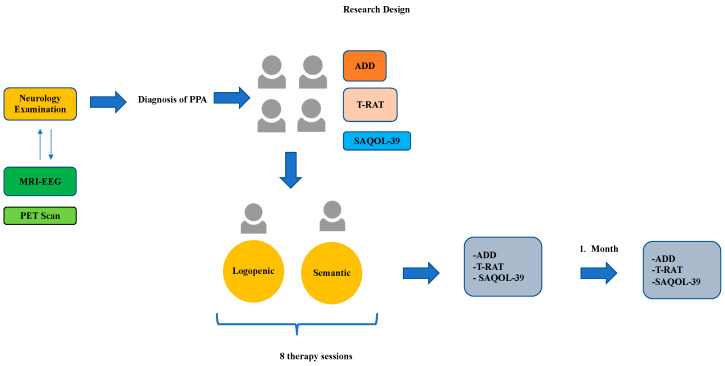
Study design.

All treatment procedures examined in this study were delivered at the University Speech and Language Therapy Unit. Following participant recruitment, a pre-test assessment was administered. The therapies were scheduled one day after the pretest. Therapy interventions were continued for 4 weeks, twice a week. The post-test was applied one day after the final therapy interventions. A follow-up test was applied one month after the therapies were completed. The speech and language therapist responsible for the pre-test, post-test, and follow-up assessments was distinct from the therapist who delivered the treatment. Demographic Information Form, Language Assessment Test for Aphasia (ADD), Stroke and Aphasia Quality of Life Scale-39 (SAQOL-39), and Turkish Picture Naming Test (T-RAT) were applied to the participants included in the study (Figure 1).

### 2.2. Tests

#### 2.2.1. Language Assessment Test for Aphasia (ADD)

Aphasia Language Assessment Test (ADD), developed by Maviş and Toğram (2009) for individuals with brain injury, serves to (a) determine performance in all language domains, (b) diagnose aphasia, and (c) facilitate the selection of appropriate therapy targets [46]. The validity, reliability, and standardization of the ADD were evaluated by Toğram and Maviş (2012) in both healthy individuals and individuals with stroke [47]. The reliability coefficients for the ADD subsections range from 0.94 to 0.99, with an overall test reliability of 0.99. The ADD comprises eight subsections evaluating various language and speech characteristics, including spontaneous language and speech, auditory comprehension, repetition, naming, reading, grammar, speech, word action, and writing. In this study, only four subsections were administered, with a maximum total score of 162, where higher scores indicate more effective language and speech skills. The spontaneous language and speech subsection includes two components: the language and cognition assessment (20 points) and the automatic speech assessment (12 points), yielding a maximum score of 32. The auditory comprehension subsection consists of command following (8 points), understanding yes/no questions (10 points), object comprehension (12 points), word/word-block level comprehension (20 points), and sentence diversity comprehension (16 points), with a maximum score of 66. The repetition subsection has a maximum score of 20, while the naming subsection is divided into categorical naming (4 points), picture-based naming (20 points), and response-based naming (20 points), resulting in a maximum score of 44. The ADD was used to measure the communication, language, and speaking skills of individuals with PPA.

#### 2.2.2. Turkish Picture Naming Test (T-RAT)

T-RAT, developed by Maviş and Tunçer (2020), is used to determine the “confrontation naming” skills of individuals with aphasia by looking at the pictures shown to them [48]. In this test, there are 150 items in total, each of which consists of colored images (a photo of the target word) on separate pages. The 150 words in this test were shown to the participants one by one, and the participant was asked, “What is this?” and was asked to name them. If participants gave incorrect answers or did not respond at all, they were given clues using “Semantic Description”, “Give Clue with Lip”, or “Phonemic Clue” and asked to rename them. The maximum was 150 points; correct answers are scored as 1 point, and incorrect or wrong answers as 0 points. The T-RAT test was used to measure picture naming skills and to evaluate related responses.

#### 2.2.3. Stroke and Aphasia Quality of Life Scale-39 (SAQOL-39)

SAQOL-39 was developed by Hilari et al. (2003) to assess the quality of life in individuals with aphasia experiencing language and speech impairments [49]. The scale was adapted into Turkish and subjected to validity and reliability analyses by Noyan (2013) [50]. The scale consists of 39 items with a 5-point Likert response format and is intended to measure quality of life. As a result of validity and reliability analyses, the Turkish version of the scale retains the following four subdomains of the original: physical (17 items), communication (7 items), psychosocial (11 items), and energy (4 items).

The scores for each item range from 1 to 5, with higher scores indicating a better quality of life and lower scores indicating a poorer quality of life. Upon completion of the scale, five separate scores are generated for its subdomains as well as an overall total score. These scores are obtained by dividing the total score for the subdomains by the number of items in that subdomain, and the total score for the entire scale is obtained by dividing the sum of all items by the total number of items in the scale. The SAQOL test was used to assess patients’ quality of life, including their language and speech skills, as well as situations related to communication and social life participation.

### 2.3. Semantic Feature Analysis Treatment

Semantic Feature Analysis was preferred as the therapy method. A verbal word–picture matching task was constructed to outline the structure and objectives of the intervention and to evaluate its effectiveness. Each item in the 100-word matching set comprised four images as follows: a single target and three distractors. Visual stimuli included photographs or color illustrations of real objects. Target stimuli were selected from the categories of clothing, objects (accessories), vehicles, body parts, and food and beverages. The main reasons for choosing these categories are that they are frequently used in daily life activities, and the words in these categories have a facilitating effect on the organization of the life activities of individuals. Word frequency and imageability (concreteness) parameters affect an individual’s naming performance. The three distractors on the target image were designed as semantic, phonological, and familiarity distractors.

During the verbal word–picture matching task, the participant was prompted to identify the target image in each set using a verbal cue (‘Show me the ______’). Images that were incorrectly identified on two or more attempts were selected for subsequent treatment.

**Figure 2:**
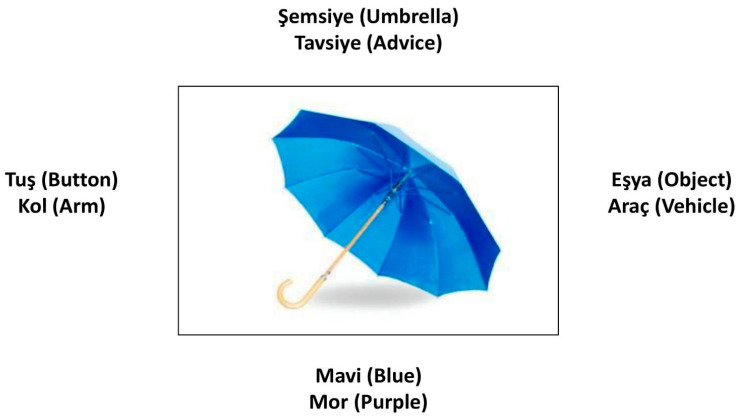
An example of semantic feature analysis used as a treatment stimulus during treatment.

Semantic Feature Analysis materials were constructed on white A4 sheets (28.1 × 22.0 cm). A large photograph or color drawing of the target item (15 × 12 cm) was positioned centrally. Four pairs of printed semantic features were arranged around the image; in each pair, one feature corresponded to the treatment target and the other functioned as a distractor (Figure 2).

There are 4 distractors written around the picture. Turkish phonetically pronounced words are close to the answers to be given for the 4 categories regarding the semantic features related to the target word. However, each participant was asked separate questions for 6 different categories, and answers were received. Distractor words for 4 of the 6 related categories were determined only for the target word (phonetically close) and were used to increase production and semantic relationships while giving clues or distracting. Semantic features were provided in paired form to promote active participation in semantic processing by prompting the participant to make accurate and informed judgments. Semantic features consisted of six options as follows: Category, Use, Action, Properties, Location, and Associations [51,52]. In the study, words with high semantic fluency related to semantic fluency were selected. Within the scope of the study, verbal fluency values were taken into account in the selection of words to be used in Semantic Features Analysis. Verbal fluency is measured by the number of words that individuals produce in a certain period of time in accordance with the desired conditions [53]. Words with high verbal fluency are more learnable. In addition, the more frequently a word is used in a language, the lower the activation threshold for that word, and the easier it will be to recall [54]. Although basically picture-level naming and relationships are studied, when the relevant categories are asked, the use in sentences is encouraged. Single-word-level response-naming with sequencing was supported by a single-word-level response for the relevant categorical semantic relationship, followed by sentence-level response sampling and sentence completion.

Twenty words were selected according to their frequency of use in daily language from the semantic fluency categories described in the work of Tunçer and Maviş and Tunçer, covering the categories of clothing, objects (accessories), vehicles, body parts, and food and beverages. Target words were selected based on high frequency in daily usage and personal relevance to ensure functional communication. Only words that participants failed to name correctly during baseline assessments were included in the list. Of the words in the list, 10 (the first 10 at the beginning of the list) were chosen with high semantic fluency (HSF), 5 (from the middle of the list) with medium semantic fluency (MSF), and 5 (at the end of the list) with low semantic fluency (LSF). The categories selected for SFA and the words related to these categories are given in Table S2.

For the following four weeks, a total of eight therapy sessions of forty minutes each were applied, two days per week. Therapy sessions were conducted by the speech and language pathologist with five years of experience. Each session followed a structured format: confrontation, naming of the target, guided feature generation using the SFA chart (group, use, action, properties, location, and association), and a final naming attempt. To ensure intervention fidelity, the therapist completed a procedural checklist after each session. No home practice was assigned to strictly control the intervention dosage. During each session, participants were asked to name the central image in each picture. For every stimulus item, the following structured prompts were administered: Category (“This is a…”), Use (“It is used for…”), Action (“What do you do with it?”), Properties (“Describe its features”), Location (“Where is it used?”), and Relationship (“It reminds me of…”).

If the participant remained unable to name the image, repetition was requested. During target concept naming and semantic network construction, cueing was provided to facilitate verbalization of the concept’s semantic features. Once the target concept was named, the clinician recorded the relevant semantic features in the corresponding boxes and guided the participant in constructing appropriate sentence structures.

### 2.4. Treatments

#### 2.4.1. Pretreatment

ADD, T-RAT, and SAQOL-39 assessments were administered to all participants one day before the therapy session, and the obtained scores were documented.

#### 2.4.2. Treatment

The abovementioned SFA-related protocols were applied.

#### 2.4.3. Post-Treatment

ADD, T-RAT, and SAQOL-39 assessments were repeated one day after completion of the therapy. One month after the treatment phase, a follow-up evaluation was conducted to assess the durability of naming and language improvements after treatment ended.

### 2.5. Statistical Analysis

Statistical analysis was performed using SPSS version 20 statistical software. The Shapiro–Wilk test was used to evaluate the normal distribution of the measured data. Median, standard deviation, minimum, and maximum values of continuous variables, and n and percentage values of categorical variables are given. Student’s *t*-test was used for the analysis of normally distributed data in the comparison between groups and the Mann–Whitney U test was used for data that did not show normal distribution. To compare repeated measures, repeated measures ANOVA was used for normally distributed data, and Friedman’s test was used for non-normally distributed data. If there was a difference between the measurements, the Wilcoxon test was used for pairwise comparison. The significance value for all statistics was determined as *p* < 0.05.

## 3. Results

Fourteen participants with PPA, seven of whom had logopenic and seven who had semantic, were included in the study. There were four women and three men in both the logopenic and semantic groups, and six of the participants in both groups were married, and one was single. Five of the logopenic participants and six of the semantic participants are working. While five of the logopenic participants had left posterior perisylvian atrophy and two had left parietal atrophy, all the semantic participants had anterior temporal lobe atrophy. The mean age of the logopenic participants was 52.85 ± 1.95 years, and the mean time post-onset was 5.85 ± 1.06 months, while the mean age of the semantic participants was 51.57 ± 2.37 years, and the mean time post-onset was 5.28 ± 1.49 months.

The ADD, T-RAT, and SAQOL-39 test results of the participants are given in Table 2. In the pretest performed before therapy, the ADD total score was 98.57 ± 7.43, the T-RAT total score was 96.64 ± 25.94, and the SAQOL-39 total score was 105.42 ± 3.91. In the post-test performed one day after the therapy, the ADD total score increased to 136.14 ± 7.93, the T-RAT total score increased to 123.64 ± 18.59, and the SAQOL-39 total score increased to 125.57 ± 6.38, and these values were statistically significant compared to the pretest results before the therapy (*p* < 0.05). In addition, scores in all subsections of SAQOL-39 and in all subsections of ADD except “Understanding commands” were significantly higher after therapies compared to pretest (*p* < 0.05). In the follow-up test performed one month after the end of the treatment, it was observed that the overall scores of T-RAT and SAQOL-39, and the physical, energy, and psychosocial scores of the subsections of SAQOL-39 decreased significantly compared to the post-test results (*p* < 0.05). Similarly, in the follow-up test performed one month after the end of the treatment, it was observed that both the ADD overall score and the Spontaneous Speech, Language and Cognition Assessment, Automatic Speech, Comprehension of Commands, Comprehension of Yes/No Questions, Comprehension of Objects, and Repetition scores of the ADD subsections decreased significantly compared to the post-test results (*p* < 0.05).

**Figure 3:**
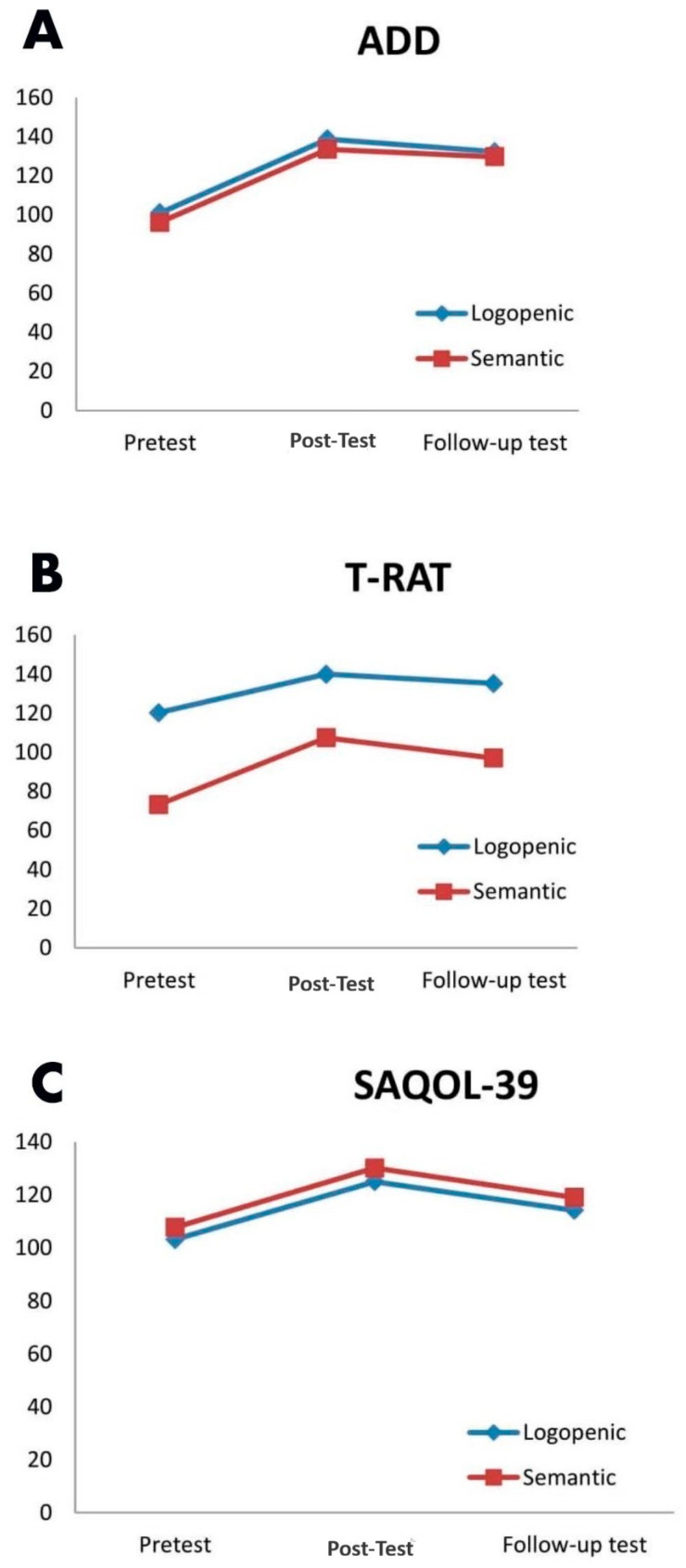
Comparison of Language Assessment Test for Aphasia (ADD) (**A**), Turkish Picture Naming Test (T-RAT) (**B**), and Stroke and Aphasia Quality of Life Scale-39 (SAQOL-39) (**C**) scores by PPA variant.

The relationship of PPA variants with ADD, SAQOL-39, and T-RAT scores before and after therapy is given in Table 3 and Figure 3.

In terms of PPA variants, no difference was observed in the ADD overall score. However, the scores of “Understanding the Categories” (*p* = 0.023) in the pretest and “Understanding Objects (*p* = 0.035) and Understanding Details from within the Categories (*p* = 0.008)” in the follow-up test were significantly higher in patients with logopenic PPA than in semantics. Both pretest and post-test T-RAT evaluation scores of those with logopenic PPA were significantly higher than those with semantic PPA (*p* < 0.001). The SAQOL-39 total scores and communication subscores of the semantic PPAs were statistically significantly higher in the pretest (*p* = 0.033) and post-test (*p* = 0.024) and numerically higher in the follow-up test than the logopenic PPAs.

## 4. Discussion

This study evaluated the effectiveness of Semantic Feature Analysis treatment in patients with semantic and logopenic PPA using ADD, SAQOL-39, and T-RAT tests. With Semantic Feature Analysis treatment applied twice a week for four weeks, ADD, SAQOL-39, and T-RAT scores increased significantly in patients with both logopenic and semantic PPA compared to pre-therapy. However, a decrease was determined in the follow-up test scores performed one month after the therapy compared to the post-test scores. T-RAT scores of patients with lvPPA were higher than those of patients with svPPA, and SAQOL-39 scores of patients with svPPA were higher than those of patients with lvPPA.

Since there are no pharmacological agents for the treatment of PPA, cognitive-linguistic treatments and interventions targeting communication can be applied. Because the interventions are on naming difficulties, a common feature of all PPA variants, semantic therapy is widely used and targeted [55]. Since the naming difficulties, especially in svPPA patients, are closely linked to deficits affecting the lexical-access system, semantic treatment is crucial to support these underlying mechanisms. In this context, the purpose of semantic intervention is to facilitate lexical retrieval by strengthening the activation of functional, perceptual, and structural features, thereby reinforcing the semantic networks. Consequently, these interventions aim to compensate for semantic degradation and facilitate successful word production [56,57,58,59]. Usually, in this treatment, auditory and/or written word-to-picture matching, yes/no questions about the target answer, and the relationship of the target words to a series of pictures are covered. In addition, target descriptions are made regarding the repetitions and the picture shown to reinforce the recall [56,60,61,62,63]. In this study, as exemplified above, tasks such as auditory and/or written word-to-picture matching, yes/no questions regarding the target, and associating target words with a series of pictures were practiced. In this study, significant improvement was observed in the ADD, T-RAT, and SAQOL-39 results of patients with both svPPA and lvPPA after SFA treatment. The results of the study reaffirm the results of previous studies showing that the therapy showed potential benefits. Henry et al. (2013) [34] applied lexical retrieval training in an individual with lvPPA and an individual with svPPA. A structured treatment protocol was designed by combining the applied therapy with semantic, spelling, and phonological information. After the therapy, it was observed that the individual with both lvPPA and svPPA consistently improved a range of target naming skills and maintained their gains. Moreover, both participants stated that they felt more confident in word recall and communication during speaking. In another similar study, the authors determined that the administered intervention was broadly beneficial for word retrieval deficits in PPA, whether due to semantic or phonological impairment. Moreover, they documented no difference between svPPA and lvPPA in terms of outcomes of lexical retrieval training. The researchers also suggested that applied lexical retrieval training has the potential to benefit most lvPPA and svPPA patients in the mild-to-moderate disease severity range [64]. Kamath et al. (2020) analyzed the responses of all three PPA variants with a meta-analysis [65]. However, T-RAT scores were found to be higher in patients with logopenic PPA in the tests performed throughout the study compared to patients with semantic PPA. It is not surprising that individuals with svPPA, who are characterized by impaired confrontational naming and single-word comprehension deficits, have lower T-RAT scores. At the same time, the SAQOL-39 total score and communication subscore were higher in individuals with svPPA than in individuals with lvPPA. In fact, the high total score is due to the large difference in the communication subscore. As a result of these scores, it can be said that communication problems in individuals with lvPPA are higher than in individuals with svPPA. The observed improvement in SAQOL-39 scores suggests that strengthening semantic networks through SFA may positively influence communication-related quality of life, potentially offering short-term benefits even within a brief intervention period. This may have led to an increase in communication and quality of life skills. It is also suggested that patients’ mood may play an important role [61]. Patients with lvPPA have been reported to exhibit anxiety, irritability, and emotional and physical attachment to their primary caregivers [66,67]. This may have resulted in lower SAQOL-39 scores in patients with lvPPA.

Although positive results of speech and language interventions for the treatment of PPA anomia have been reported, it is unclear whether gains are maintained over time. In studies conducted, results with a different follow-up period are generally reported, and research results are not consistent. However, the duration of the intervention is also important because one of the most important factors in maintaining the treatment gains is the duration of adequate treatment. In studies using lexical retrieval treatment, the duration of treatment ranged from 2 weeks [68] to 12 weeks [69]. Follow-up periods ranged from 4 weeks to 84 weeks. During the treatment period, only the participants who received SLT [68,69,70,71,72] showed improvement in the studied items and no improvement in the unstudied items. Moreover, it was determined that the gains were not preserved during the follow-up period. However, in studies in which intensive therapy was applied or homework was combined with therapy [28,35,64,73,74,75], improvement was detected in both the studied items and the unstudied items. It was also reported that the gains were maintained throughout the follow-up period. In studies where semantic treatment was applied, the duration of treatment ranged from 3 weeks [76,77,78,79,80,81] to 96 weeks [82]. Follow-up times ranged from 4 weeks to 24 weeks. In studies with more weekly sessions [17,79,81,83,84] or in studies with homework [85], improvement was determined in both worked and unstudied items. However, in studies with few weekly sessions [57,60,63,76,77,78,80,85], improvement was observed only in the items studied. In the majority of studies, it was reported that the gains were maintained throughout the follow-up period. This study had a follow-up of one month after four weeks of intervention. In the follow-up tests performed after one month, it was determined that the ADD, T-RAT, and SAQOL39 test results were mostly decreased compared to the post-test results. However, follow-up test results were still higher than pretest results. This shows that the gains obtained with treatment are partially preserved despite the decrease. These results are in agreement with the results of the other studies mentioned above.

### Limitation

This study has several limitations. First, the study was single-centered, and the number of participants was limited due to the rarity of PPA as a neurodegenerative disease, which prevented diversity and randomization. The most significant limitation is the lack of an untreated or wait-list control group. Since PPA is a progressive disorder and the test battery was highly structured, observed gains may reflect practice effects, test–retest familiarity, or non-specific engagement rather than true therapeutic change.

In addition, the intervention consisted of only eight sessions over four weeks, which is below the typical intensities used in PPA studies. While this low dose limits the interpretation of treatment effects, the session duration (40 min) and frequency were determined based on the participants’ limited attention spans and fatigue levels observed during therapy. Furthermore, due to the nature of the disease, the motivation of participants and families for recovery was low, and no feedback was obtained from caregivers. Future studies comparing different treatments with longer durations, follow-up periods, and randomized controlled designs are needed.

It is well-established in the literature that the logopenic variant (lvPPA) is often associated with underlying Alzheimer’s disease pathology. However, in the current study, biomarkers (e.g., CSF analysis) or amyloid imaging were not assessed. Therefore, the observed results should be interpreted as clinical outcomes rather than establishing a direct link to specific underlying etiologies.

Additionally, due to the small sample size, effect sizes were not calculated to avoid potential bias or overestimation of the treatment magnitude. Future studies with larger cohorts are needed to provide more reliable estimates of treatment effects.

## 5. Conclusions

In this study, we show that the modified Semantic Feature Analysis treatment intervention that we applied was effective in individuals with both svPPA and lvPPA. The findings of this study add to the growing body of evidence from other studies supporting the benefit of speech-language intervention for individuals with PPA. It is seen that therapy approaches based on semantic relationships and production skills, which include the naming processes of cases with PPA whose mother tongue is Turkish, are beneficial. Although it is a degenerative disease, it is very important to include it in therapy processes with an approach based on semantic restoration in individuals with PPA. This situation should not be limited to individuals whose native language is Turkish, and it is recommended to conduct similar therapy content and studies in different languages and cultures. Longer-term and larger population studies are needed to establish a proven treatment protocol for patients with PPA.

## Figures and Tables

**Table 1:** Demographic and clinical characteristics of the participants.

Groups	Gender	Age (Year)	Educational Status	Employment Status	MRI Damage Area	Onset Time (Months)	Dominant Hand	Marital Status
Logopenic1	F	52	High School	Yes	LPPA	7	Right	Single
Logopenic2	M	55	University	Yes	LPPA	5	Right	Married
Logopenic3	F	56	High School	No	LPPA	6	Right	Married
Logopenic4	M	53	University	Yes	LPPA	7	Right	Married
Logopenic5	M	51	High School	Yes	LPA	6	Right	Married
Logopenic6	F	52	University	Yes	LPA	6	Right	Married
Logopenic7	F	51	High School	No	LPPA	4	Right	Married
Semantic1	M	51	High School	Yes	ATLA	5	Right	Married
Semantic2	M	54	High School	Yes	ATLA	7	Right	Married
Semantic3	F	55	High School	Yes	ATLA	5	Right	Married
Semantic4	F	52	University	Yes	ATLA	6	Right	Single
Semantic5	F	48	High School	No	ATLA	4	Right	Married
Semantic6	F	50	High School	Yes	ATLA	3	Right	Married
Semantic7	M	51	University	Yes	ATLA	7	Right	Single

MRI, Magnetic Resonance Imaging; LPA, Left Parietal Atrophy; LPPA, Left Posterior Perisylvian Atrophy; ATLA, Anterior Temporal Lobe Atrophy.

**Table 2:** Comparison of Language Assessment Test for Aphasia (ADD), Turkish Picture Naming Test (T-RAT), and Stroke and Aphasia Quality of Life Scale-39 (SAQOL-39) scores applied to the participants. Results are given as mean ± S.D median (min–max) demographic and clinical characteristics of the participants.

Pretest	Posttest	Follow-Up Test	
ADD			
17.85 ± 1.29	18.92 ± 0.82 ^a^	18.21 ± 0.97 ^b^	Spontaneous Speech, Language, and Cognition Assessment
18 (16–20)	19 (18–20)	18 (16–20)	
10.50 ± 1.01	11.28 ± 0.72 ^a^	10.92 ± 0.82 ^a. b^	Spontaneous speech
11 (8–12)	11 (10–12)	11 (9–12)	
7.64 ± 0.49	7.85 ± 0.36	7.42 ± 0.75 ^b^	Understanding commands
8 (7–8)	8 (7–8)	8 (6–8)	
8.50 ± 0.51	9.57 ± 0.75 ^a^	9.28 ± 0.91 ^a. b^	Understanding Yes/No questions
8.5 (8–9)	10 (8–10)	9.5 (7–10)	
978 ± 1.36	11.07 ± 1.07 ^a^	10.64 ± 1.15 ^a. b^	Understanding Objects
9.5 (8–12)	11.5 (9–12)	11 (9–12)	
8.57 ± 0.93	9.28 ± 0.82 ^a^	9.14 ± 0.86	Understanding the Categories
8 (7–10)	9.5 (8–10)	9 (8–10)	
7.78 ± 1.05	8.78 ± 1.05 ^a^	8.64 ± 0.92 ^a^	Understanding the details within the category
8 (6–10)	8.5 (7–10)	8.5 (7–10)	
6.35 ± 1.33	7.50 ± 0.75 ^a^	7.42 ± 0.85 ^a^	Simple sentence matching
7 (4–8)	8 (6–8)	8 (5–8)	
5.78 ± 1.12	6.92 ± 1.14 ^a^	6.78 ± 1.18 ^a^	Complex sentence matching
6 (4–8)	7 (4–8)	7 (4–8)	
10.00 ± 1.00	16.07 ± 1.77 ^a^	15.35 ± 2.30 ^a. b^	Repetition
10 (1–18)	16 (13–18)	16 (11–18)	
0.50 ± 0.65	2.35 ± 1.44 ^a^	2.14 ± 1.23 ^a^	Categorical Naming
0 (0–5)	2.5 (0–4)	2.5 (0–4)	
2.71 ± 2.52	13.64 ± 4.25 ^a^	12.85 ± 3.52 ^a^	Naming by looking at the picture
2 (0–7)	13.5 (6–20)	12.5 (6–18)	
1.00 ± 0.78	6.71 ± 1.81 ^a^	6.14 ± 1.83 ^a^	Noun naming
1 (0–2)	6.5 (3–10)	6.5 (3–9)	
1.57 ± 1.22	6.14 ± 2.65 ^a^	6.00 ± 2.82 ^a^	Action naming
1 (0–3)	6 (2–10)	6 (1–10)	
98.57 ± 7.43	136.14 ± 7.93 ^a^	131.00 ± 8.62 ^a. b^	ADD total score
99 (85–111)	135 (120–150)	13.05 (119–146)	
T-RAT			
96.64 ± 25.94	123.64 ± 18.59 ^a^	116.07 ± 20.87 ^a. b^	
98.50 (56–134)	125 (96–148)	119 (90–142)	
SAQOL-39			
66.78 ± 1.36	71.07 ± 2.33 *	63.21 ± 3.82 * ^‡^	Physical
10.00 ± 1.30	13.21 ± 1.76 *	12.00 ± 1.70 * ^‡^	Energy
20.85 ± 1.99	30.42 ± 4.05 *	28.57 ± 3.91 * ^‡^	Psychosocial
7.78 ± 2.60	12.85 ± 3.03 *	12.78 ± 2.66 *	Communication
105.42 ± 3.91	125.57 ± 6.38 *	116.57 ± 6.36 * ^‡^	Total score

Friedman test and Wilcoxon test; *p* < 0.05 compared to ^a^ pretest, *p* < 0.05 compared to ^b^ post-test. Repeated measures ANOVA; *p* < 0.05 compared to * pretest, *p* < 0.05 compared to ^‡^ posttest.

**Table 3:** Comparison of pre- and post-treatment scores of the Language Assessment Test for Aphasia (ADD), Turkish Picture Naming Test (T-RAT), and Stroke and Aphasia Quality of Life Scale-39 (SAQOL-39) for PPA variants.

		Pretest		Posttest		Follow-Up Test	
	Gender	Mean ± S.D.	Z/t. p	Mean ± S.D.	Z/t. p	Mean ± S.D.	Z/t. p
ADD							
Spontaneous Speech, Language, and Cognition Assessment	Female	17.12 ± 0.83	Z = −2.385	18.62 ± 0.74	Z = −1.574	17.75 ± 0.88	Z = −2.164
	Male	18.83 ± 1.19	*p* = 0.017	19.33 ± 0.81	*p* = 0.116	18.83 ± 0.75	*p* = 0.030
Spontaneous speech	Female	10.87 ± 0.64	Z = −1.366	11.37 ± 0.74	Z = −0.562	11.00 ± 0.53	Z = −0.072
	Male	10.00 ± 1.26	*p* = 0.172	11.16 ± 0.75	*p* = 0.574	10.83 ± 1.16	*p* = 0.943
Understanding commands	Female	7.62 ± 0.51	Z = −0.155	7.87 ± 0.35	Z = −0.212	7.50 ± 0.75	Z = −0.435
	Male	7.66 ± 0.51	*p* = 0.877	7.83 ± 0.40	*p* = 0.832	7.33 ± 0.81	*p* = 0.663
Understanding Yes/No questions	Female	8.75 ± 0.46	Z = −2.082	9.87 ± 0.35	Z = −1.623	9.62 ± 0.51	Z = −1.415
	Male	8.16 ± 0.40	*p* = 0.037	9.16 ± 0.98	*p* = 0.105	8.83 ± 1.16	*p* = 0.157
Understanding Objects	Female	10.50 ± 1.30	Z = −2.214	11.37 ± 0.91	Z = −1.188	10.87 ± 0.99	Z = −0.800
	Male	8.83 ± 0.75	*p* = 0.027	10.66 ± 1.21	*p* = 0.235	10.33 ± 1.36	*p* = 0.424
Understanding the Categories	Female	9.00 ± 0.92	Z = −1.950	9.50 ± 0.75	Z = −1.123	9.25 ± 0.88	Z = −0.551
	Male	8.00 ± 0.63	*p* = 0.051	9.00 ± 0.89	*p* = 0.262	9.00 ± 0.89	*p* = 0.582
Understanding the details within the category	Female	8.37 ± 0.91	Z = −2.571	9.25 ± 0.88	Z = −1.930	8.75 ± 0.88	Z = −0.410
	Male	7.00 ± 0.63	*p* = 0.010	8.16 ± 0.98	*p* = 0.054	8.50 ± 1.04	*p* = 0.682
Simple sentence matching	Female	6.75 ± 1.16	Z = −1.384	7.87 ± 0.35	Z = −2.122	7.37 ± 1.06	Z = −0.220
	Male	5.83 ± 1.4	*p* = 0.166	7.00 ± 0.89	*p* = 0.034	7.50 ± 0.54	*p* = 0.825
Complex sentence matching	Female	6.00 ± 1.30	Z = −0.744	6.87 ± 1.45	Z = −0.272	6.50 ± 1.41	Z = −0.817
	Male	5.50 ± 0.83	*p* = 0.457	7.00 ± 0.63	*p* = 0.786	7.16 ± 0.75	*p* = 0.414
Repetition	Female	4.87 ± 4.38	Z = −2.986	15.00 ± 1.51	Z = −2.779	13.87 ± 1.88	Z = −2.819
	Male	16.83 ± 1.60	*p* = 0.003	17.50 ± 0.83	*p* = 0.005	17.33 ± 0.81	*p* = 0.005
Categorical Naming	Female	0.62 ± 0.74	Z = −0.735	2.25 ± 1.75	Z = −0.132	1.87 ± 1.35	Z = −0.744
	Male	0.33 ± 0.51	*p* = 0.462	2.50 ± 1.04	*p* = 0.895	2.50 ± 1.04	*p* = 0.457
Naming by looking at the picture	Female	3.37 ± 2.32	Z = −1.314	11.37 ± 2.97	Z = −2.207	10.62 ± 2.44	Z = −2.741
	Male	1.83 ± 2.71	*p* = 0.189	16.66 ± 3.93	*p* = 0.027	15.83 ± 2.31	*p* = 0.006
Noun Naming	Female	1.12 ± 0.8	Z = −0.688	6.25 ± 2.05	Z = −1.249	5.62 ± 1.59	Z = −1.511
	Male	0.83 ± 0.75	*p* = 0.491	7.33 ± 1.36	*p* = 0.212	6.83 ± 2.04	*p* = 0.131
Action naming	Female	2.37 ± 0.91	Z = −2.853	4.87 ± 2.35	Z = −2.079	4.62 ± 2.55	Z = −2.144
	Male	0.50 ± 0.54	*p* = 0.004	7.83 ± 2.13	*p* = 0.038	7.83 ± 2.13	*p* = 0.032
Total	Female	97.37 ± 8.78	Z = −0.974	132.37 ± 5.78	Z = −1.945	125.25 ± 4.77	Z = −2.840
	Male	100.16 ± 5.52	*p* = 0.330	141.16 ± 7.98	*p* = 0.052	138.66 ± 6.18	*p* = 0.005
T-RAT							
	Female	97.00 ± 30.64	t = 0.057	122.62 ± 22.36	t = −0.228	115.00 ± 24.46	t = −0.213
	Male	96.16 ± 20.85	*p* = 0.955	125.00 ± 13.97	*p* = 0.824	117.50 ± 17.06	*p* = 0.835
SAQOL-39							
Physical	Female	66.62 ± 1.40	Z = −0.473	71.12 ± 2.53	Z = −0.200	62.62 ± 3.33	Z = −0.523
	Male	67.00 ± 1.41	*p* = 0.636	71.00 ± 2.28	*p* = 0.841	64.00 ± 4.60	*p* = 0.601
Energy	Female	9.62 ± 1.50	Z = −1.179	12.50 ± 1.41	Z = −1.714	11.62 ± 1.40	Z = −0.983
	Male	10.50 ± 0.83	*p* = 0.238	14.16 ± 1.83	*p* = 0.086	12.50 ± 2.07	*p* = 0.325
Psychosocial	Female	20.62 ± 1.92	Z = −0.468	29.50 ± 3.50	Z = −0.917	27.62 ± 3.77	Z = −0.842
	Male	21.16 ± 2.22	*p* = 0.640	31.66 ± 4.71	*p* = 0.359	29.83 ± 4.07	*p* = 0.400
Communication	Female	7.62 ± 2.77	Z = −0.197	12.37 ± 3.15	Z = −0.716	11.87 ± 2.47	Z = −1.583
	Male	8.00 ± 2.60	*p* = 0.844	13.50 ± 3.01	*p* = 0.474	14.00 ± 2.60	*p* = 0.118
Total score	Female	104.50 ± 4.07	Z = −0.391	125.50 ± 4.50	Z = −0.782	113.75 ± 4.23	Z = −1.624
	Male	106.66 ± 3.66	*p* = 0.696	130.33 ± 7.84	*p* = 0.434	120.33 ± 7.11	*p* = 0.104

## Data Availability

The data that support the findings of this study are available from the corresponding author upon reasonable request.

## Supplementary Materials

The following supporting information can be downloaded at: https://www.mdpi.com/article/10.3390/healthcare14020272/s1. Table S1: Demographic information; Table S2: Items and words chosen for the SFA; Table S3: Inter-observer agreement values.

## Abbreviations

The following abbreviations are used in this manuscript:

PPA	Primary Progressive Aphasia
svPPA	Semantic Primary Progressive Aphasia
nfvPPA	Nonfluent/Agrammatic Primary Progressive Aphasia
lvPPA	Logopenic Primary Progressive Aphasia
ADD	Language Assessment Test for Aphasia
T-RAT	Turkish Picture Naming Test
SAQOL-39	Stroke and Aphasia Quality of Life Scale-39
SFA	Semantic Feature Analysis
HSF	High Semantic Fluency
MSF	Medium Semantic Fluency
LSF	Low Semantic Fluency
