# Supplementary material for: Treatment Efficacy of Semantic Feature Analysis in Logopenic and Semantic Variants of Primary Progressive Aphasia
Source: Healthcare (Basel). 2026 Jan 21;14(2):272. doi: 10.3390/healthcare14020272 (PMC12841384; doi:10.3390/healthcare14020272)
Supplement: Supplementary file 1 [file healthcare-14-00272-s001.zip › healthcare-4027838-supplementary.pdf]

Table S1. Demographic information

ID	1. Gender	2. Year of Birth	3.Educational status	4. Pre-diagnostic working status	5. Job	6. Marital status	7. Who do you live with?	8. Aphasia Type	8.1 Radiological Imaging	8.2 Damage Area	9. Diagosis of aphasia	10. Status of receiving aphasia treatment	10.1 Therapy History	11. When?	12. Dominant hand	13.Psychiatric consultation-treatment	14. Additional neurological diagnosis
L1	F	1970	High School	Yes	Officer	Single	Family	Logopenic (PPA)	MRI and PET Scan	left posterior perisylvian atrophy	7 months ago	No			Right	No	None
L2	M	1967	University	Yes	Engineer	Married	Family	Logopenic (PPA)	MRI and PET Scan	left posterior perisylvian atrophy	5 months ago	No			Right	No	None
L3	F	1966	High School	No	Housewife	Married	Family	Logopenic (PPA)	MRI and PET Scan	left posterior perisylvian atrophy	6 months ago	No			Right	Yes	None
L4	M	1969	University	Yes	Banker	Married	Family	Logopenic (PPA)	MRI and PET Scan	left posterior perisylvian atrophy	7 months ago	Yes- 3 months ago	1 session per week - for 2 months"	3 month s ago	Right	Yes	None
L5	M	1971	High School	Yes	Textile	Married	Family	Logopenic (PPA)	MRI and PET Scan	left parietal atrophy	6 months ago	No			Right	No	None
L6	F	1970	Vocational School	Yes	Worker	Married	Family	Logopenic (PPA)	MRI and PET Scan	left parietal atrophy	6 months ago	No			Right	No	None
L7	F	1971	High School	No	Housewife	Married	Family	Logopenic	MRI and PET	left posterior	4 months	No			Right	No	None

								(PPA)	Scan	perisylvian atrophy	ago						
S1	M	1971	High School	Yes	Officer	Married	Family	Semantic (PPA)	MRI and PET Scan	anterior temporal lobe atrophy	5 months ago	No			Right	Yes	None
S2	M	1968	High School	Yes	Officer	Married	Family	Semantic (PPA)	MRI and PET Scan	anterior temporal lobe atrophy	7 months ago	No			Right	No	None
S3	F	1967	High School	Yes	Worker	Married	Family	Semantic (PPA)	MRI and PET Scan	anterior temporal lobe atrophy	5 months ago	No			Right	No	None
S4	F	1970	University	Yes	Academician	Single	Family	Semantic (PPA)	MRI and PET Scan	anterior temporal lobe atrophy	6 months ago	No			Right	No	None
S5	F	1974	High School	No	Housewife	Married	Family	Semantic (PPA)	MRI and PET Scan	anterior temporal lobe atrophy	4 months ago	Yes- 2 months ago	2 session per week - for 4 weeks	2 months ago	Right	No	None
S6	F	1972	High School	Yes	Artisan	Married	Family	Semantic (PPA)	MRI and PET Scan	anterior temporal lobe atrophy	3 months ago	No					
S7	M	1971	University	Yes	Business manager	Married	Family	Semantic (PPA)	MRI and PET Scan	anterior temporal lobe atrophy	7 months ago	No					

Table S2. Items and words chosen for the SFA

Clothes		Frequency	Objects		Frequency	Vehicles		Frequency	Parts of body		Frequency	Food-Drinks		Frequency
Socks	Çorap	HF	Scarf	Atkı	HF	Airplane	Uçak	HF	Nose	Burun	HF	Börek	Börek	HF
Shirt	Gömlek	HF	Newspaper	Gazete	HF	Bus	Otobüs	HF	Eye	Göz	HF	Soup	Çorba	HF
Jacket	Ceket	HF	Blanket	Battaniye	HF	Train	Tren	HF	Foot	Ayak	HF	Meatball	Köfte	HF
Shoe	Ayakkab ı	HF	Watch	Saat	HF	Truck	Kamyon	HF	Ear	Kulak	HF	Chicken	Tavuk	HF
Pullover	Kazak	HF	Wallet	Cüzdan	HF	Taxi	Taksi	HF	Arm	Kol	HF	Salad	Salata	HF
Skirt	Etek	HF	Calendar	Takvim	HF	Ship	Gemi	HF	Leg	Bacak	HF	Bread	Ekmek	HF
Pyjamas	Pijama	HF	Pillow	Yastık	HF	Bicycle	Bisiklet	HF	Hand	El	HF	Tea	Çay	HF
Cardigan	Hırka	HF	Phone	Telefon	HF	Car	Araba	HF	Finger	Parmak	HF	Cheese (White)	Peynir (Beyaz)	HF
Slipper	Terlik	HF	Glasses	Gözlük	HF	Minibus	Minibüs	HF	Hair	Saç	HF	Tomato	Domate s	HF
Glove	Eldiven	HF	Para	Para	HF	Tram	Tramvay	HF	Tongue	Dil	HF	Water	Su	HF
Tie	Kravat	MF	Ring	Yüzük (Alyans)	MF	Boat	Kayık	MF	Shoulder	Omuz	MF	Pasta	Pasta	MF
Beanie	Bere	MF	Umbrella	Şemsiye	MF	Tractor	Traktör	MF	Ankle	Bilek	MF	Pickle	Turşu	MF
Shawl	Şal	MF	Frame	Çerçeve	MF	Subway	Metro	MF	Brain	Beyin	MF	Egg	Yumurt a	MF
Boat	Bot	MF	Idendity Card	Kimlik (Nüfus Cüzdanı)	MF	Baloon	Balon	MF	Lung	Ciğer	MF	Lemonade	Limona ta	MF
Belt	Kemer	MF	Handkerchie f	Mendil	MF	Metrobus	Metrobüs	MF	Lip	Dudak	MF	Bagel	Simit	MF
Fur	Kürk	LF	Letter	Mektup	LF	Phaeton	Fayton	LF	Bone	Kemik	LF	Sausage	Sosis	LF
Wedding dress	Gelinlik	LF	Flag	Bayrak	LF	Ambulance	Ambulans	LF	Heel	Topuk	LF	Toast	Tost	LF
Bracelet	Bilezik	LF	Book	Kitap	LF	Digger	Kepçe	LF	Moustache	Bıyık	LF	Mantı	Mantı	LF
Bathrobe	Bornoz	LF	Computer	Bilgisayar	LF	Tank	Tank	LF	Beard	Sakal	LF	Oralet	Oralet	LF
Apron		LF	Nail	Çivi	LF	Elevator	Asansör	LF	Blood	Kan	LF	Menemen	Menem en	LF

HF: High Frequency, **MF:** Moderate Frequency, **LW:** Low Frequency

Table S3. Inter-observer agreement values

Test	Before Intervention	After Intervention	One month After Intervention
**ADD -Speech Fluency**	%100	%100	%100
**ADD -Auditory Comprehension**	%100	%100	%100
**ADD -Repetition**	%100	%100	%100
**ADD -Naming**	%100	%100	%100
**Quality of Life -Physical**	%100	%100	%100
**Quality of Life -Communication**	%100	%100	%100
**Quality of Life -Psychosocial**	%100	%100	%100
**Quality of Life -Energy**	%100	%98	%97
**T-RAT (109 Words)**	%100	%100	%100
**T-RAT (41 Words)**	%99	%100	%100
**T-RAT (150 Words)**	%99	%100	%100
